# Simulating Notch-Dome Morphology of Action Potential of Ventricular Cell: How the Speeds of Positive and Negative Feedbacks on Transmembrane Voltage Can Influence the Health of a Cell?

**DOI:** 10.1155/2020/5169241

**Published:** 2020-09-03

**Authors:** S. H. Sabzpoushan, A. Ghajarjazy

**Affiliations:** Department of Biomedical Engineering, Iran University of Science and Technology (IUST), Tehran 16846-13114, Iran

## Abstract

Ventricular action potential is well-known because of its plateau phase with a spike-notch-dome morphology. As such, the morphology of action potential is necessary for ensuring a correct heart functioning. Any distraction from normal notch-dome morphology may trigger a circus movement reentry in the form of lethal ventricular fibrillation. When the epicardial action potential dome propagates from a site where it is maintained to regions where it has been lost, it gives rise to the proposed mechanism for the Brugada syndrome. Despite the impact of notch-dome dynamics on the heart function, no independent and explicit research has been performed on the simulation of notch-dome dynamics and morphology. In this paper, using a novel mathematical approach, a three-state variable model is proposed; we show that our proposed model not only can simulate morphology of action potential of ventricular cells but also can propose a biological reasonable tool for controlling of the morphology of action potential spike-notch-dome. We show that the processes of activation and inactivation of ionic gating variables (as positive or negative feedbacks on the voltage of cell membrane) and the ratio of their speeds (time constants) can be treated as a reasonable biological tool for simulating ventricular cell notch-dome. This finding may led to a new insight to the quantification of the health of a ventricular cell and may also propose a new drug therapy strategy for cardiac diseases.

## 1. Introduction

The so-called process, “excitation-contraction coupling” in the cardiac myocyte, is unique in that it is responsible for the coupling of electrical impulse to mechanical function. There are several ions, ion channels, and regulatory pathways participating in the generation of action potential (AP) of cardiac cells. Ionic channels are large transmembrane proteins having aqueous pores through which ions can flow down their electrochemical gradients. The dynamics of electrical conductance of an individual channel is controlled by its gate dynamics, i.e., gates' speed. The gating dynamics influence the time course of the channel opening and closing [1].

The speed of gates, i.e., their time constants, ionic concentrations, membrane voltage following time sequence, and various regulatory pathways determine the morphology of AP, which can be expressed as mathematical formalisms, making it feasible to use the computational approach to analyze and elucidate the underlying mechanisms of the whole cardiac cell.

**Figure 1:**
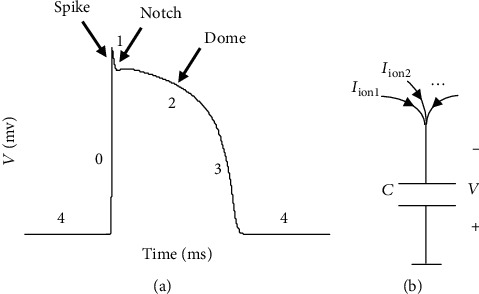
(a) A typical morphology of ventricular AP with five distinct phases and spike-notch-dome (SND) as a significant part of an AP [2]; (b) membrane voltage of a cell can be modeled by a capacitor that is charged by ionic currents.

Ventricular AP is famous because of its plateau phase with a spike-notch-dome (SND) morphology [3]. As such, the morphology of AP like Figure 1 is necessary for ensuring a correct heart functioning.

AS stated beforehand, APs are produced as a result of ion currents that cross the cell membrane via ionic channels and gates. Ion currents produce a net depolarization or repolarization of the membrane as different currents are invoked in response to the transmembrane voltage changes. Each type of channel is highly selective for a specific type of ion. The most common intracellular ion concentrations considered in cardiac models are sodium, calcium and potassium.

Channelopathies due to mutations that modify the ion channel function can perturb the form of the action potential, sometimes leading to cardiac dysfunctions or altered AP morphology and propagation [4, 5].

Following the pioneering mathematical description of the AP by Hodgkin and Huxley (HH) in neuronal cells [6], the first cardiac models considered in much the same way. The changes in the transmembrane potential,$\;\dot{v}$, produced by a sum of ionic-gated currents, ∑_ion_*I*_ion_, that is [2, 7],

(1) $$\begin{array}{c} c\dot{v}=I_{\text{stim}}-\underset{\text{ion}}{\sum }I_{\text{ion}}, \end{array}$$

where *v* and *c* are membrane's voltage and capacitance, respectively, and *I*_stim_ is stimulus current. Equation (1) that is a general formalism for modeling cell's membrane potential is illustrated schematically in Figure 1(b).In this context, the inward ionic currents that increase cell's voltage are considered negative currents and outward ones as positive currents.

Today, as more developed experimental data of the different currents and their dynamics became available, more complex models of the AP for specific cardiac cells have been proposed [8].The mission of these models has been noticeable for several animal kinds, such as guinea pig [9], dog [10], rabbit [11], or rat [12], and also for humans [2, 13–15].

The general drawback of the above electrophysiological models is the increasing complexity of the models and consequently the very high computational costs associated with the simulation of cardiac tissue as well as the difficulties and sometimes impracticality of model analyzing. Clearly, a highly realistic ionic model is ideal as an *in silico* experimental tool, allowing to safely examine multiple hypotheses and making predictions without experiencing any cardiac danger for the patient or to check conditions not easily reproducible in clinical experiments. But, despite their more realistic description, these models usually are often difficult to analyze mathematically and deducing biology principles from them and sometimes less compatible with the pathology of cardiac cells.

Simplified models have also been proposed for modeling AP dynamics. However, although they are simple, they are not complete electrophysiological [16–18], so-called semielectrophysiological models [19–23], i.e., somewhere between the simple and the complex electrophysiological models. These semiphysiological models preserve particular sets of properties of the action potential, like AP duration (APD); that is, the time during which the voltage is above a certain threshold, characterizing the duration of the excited state, or the conduction velocity (CV) [24]. In this respect, these can be viewed as mesoscopic models that bridge the gap between dynamics at the molecular level (ion channel gating) and whole macroscopic system, heart. These models are still manageable for both computation and theoretical analysis.

An example of a semiphysiological model is the one by Fenton and Karma [22], a three-state variables model of the cardiac action potential. This model uses three transmembrane currents, i.e., fast inward, slow inward, and slow outward, which resemble the set of physiological sodium, calcium, and potassium currents, respectively. In the Fenton-Karma model, the state variables are the transmembrane potential and two gating variables that are used to regulate the inactivation of the fast inward and slow inward currents, respectively.

Despite the Fenton-Karma model simplicity, the model is less compatible with electrophysiology of cardiac cell in the sense that it uses discrete functions, Heaviside function. Although the Fenton-Karma model can reproduce fairly the action potential of more complex models or experiments, on one hand, it fails to reproduce accurately the SND or notch-dome (ND) pattern and on the other hand, it has no independent and appropriate parameters for regulating ND. In fact, the Fenton-Karma model was originally created in order to describe the propagation and properties of scroll waves in cardiac tissue. Wave propagation behavior in the cardiac tissue is determined by the restitution and alternans properties of cells. With the alternans, we mean a beat to beat modification in the APD at fast pacing rates, where the onset and subsequent evolution of alternans has been related to the shape of the restitution curves [25]. However, more recent results have shown that models with the same restitution properties, but different AP morphologies, may give different onsets for alternans [21]. The reason is that the dynamics of alternans is also affected by ionic currents that depend significantly on the form of the AP [26]. Furthermore, ionic currents are also a basis factor for the occurrence of reexcitation and phase 2 reentry in cases where the duration of the action potential is nonhomogeneous in the heart tissue [27].

A modification of the three-variable Fenton-Karma model has been proposed in [19]; it fits better the AP morphology. The modified model includes an additional gate that modulates the slow inward current, in a fashion that resembles the effect of the fast outward potassium current in electrophysiological models. This allows to reproduce the spike in phase 1 of the AP typical of epicardial cells [28]. Bueno et al.'s model is still less compatible with the electrophysiology of ventricular cell because it uses the Heaviside function.

Peñaranda et al. [29] included the effect of *I*_to_, fast outward potassium current; following the same idea as in the Fenton-Karma model, they divided the currents not by their carrier ion, but by their function: inward or outward currents, and among these, slow and fast currents to a total of four. With this assumption, they showed that it is possible to reproduce the important characteristics of AP propagation and morphology. They also used their model to study reexcitations in tissue presenting large dispersion of repolarization. There, it was shown that reexcitation is due to a slow pulse induced by the L-type calcium current and propagates from the region of long APDs to the region of short APDs until it encounters newly excitable tissue.

Channelopathies are diseases that appear because of defects in ion channels; this defection causes by either genetic or acquired factors. Mutations in genes encoding ion channels, which damage channel function, are the most common cause of channelopathies. Because ion channels are greatly involved in cells, ion channel defects have been involved in a wide variety of diseases, including epilepsy, migraine, blindness, deafness, diabetes, hypertension, cardiac arrhythmia, asthma, irritable bowel syndrome, and cancer [30].

Cardiac channelopathies are responsible for many sudden arrhythmic death syndrome cases. Mutations in calcium, sodium, potassium, and TRP channel genes have been identified to cause a variety of cardiac arrhythmic disorders like Brugada syndrome types, dilated cardiomyopathy, and long QT syndrome types [31].

Although a great number of researches have been conducted in ventricular cell AP modeling, approximately all of them have paid less independent and direct attention to ND simulations and dynamics. In fact, there is nothing in electrophysiology literature connecting ND-related diseases to channelopathies.

Grandi et al. [13] developed a detailed mathematical model for Ca handling and ionic currents in the human ventricular myocyte with the main aim to simulate basic excitation–contraction coupling phenomena; their model relies on the framework of a rabbit myocyte model. They could simulate AP morphology of epicardial cells with characteristic spike and dome (SD).

AP dynamics are influenced by its morphology [21, 32, 33]. In a research by Bueno-Orovio et al. [19], they used previously published data [34–42] and developed a minimal model of the APs of human ventricular myocytes; they modified the three-variable model proposed by Fenton and Karma [22]. They argued that a three-variable formulation is not sufficient to reproduce SND morphologies. Therefore, they add a fourth variable to obtain a more accurate AP morphology with SND. However, as we will see later, although our proposed model is a three-state variable one, it can simulate SND morphology satisfactorily. It means that our proposed model can be more computational cost-effective than Bueno-Orovio et al.'s model.

In recent years, a dynamic known as phase-2 reentry (P2R) has been recognized as a mechanism for triggering lethal arrhythmias. P2R occurs when adjacent cells undergo dramatic action-potential (AP) shortening from a normal ND morphology to a loss-of-dome morphology. Local reexcitation ensues when ionic current during the AP dome stage propagates from depolarized sites to hyperpolarized loss-of-dome sites. It is thought that P2R could degenerate into ventricular fibrillation and cause sudden cardiac death.

Human AP in the Brugada syndrome have been characterized by delayed or even complete loss of dome formation, especially in the right ventricular epicardial layers. It is shown that under the condition of fast pacing rates, there is alternation between “loss of dome” and “coved dome” in the AP morphology for the epicardial cells. Peñaranda et al. in their paper presented a study on Brugada syndrome, where the loss of the dome is achieved by changing the conductance of a current in their model. Bueno et al. showed that, in one-dimensional cables, P2R can be induced by adjoining lost-dome and delayed-dome regions, as mediated by tissue excitability and transmembrane voltage profiles, and reduced coupling facilitates its induction. In two and three dimensions, sustained reentry can arise when three regions (delayed-dome, lost-dome, and normal epicardium) are present [19]. Ten et al. in their model for human ventricular cell demonstrated that the AP shows the characteristic SND architecture found for epicardial cells [2]. Epicardial and M cells, but not endocardial cells, display action potentials with a notched or SND morphology. They showed that a further shift in the balance of currents leads to the loss of the action potential dome at some epicardial sites, which is manifested in the ECG as a further ST segment elevation. Antzelevitch showed that because the loss of the action potential dome in the epicardium is generally not spatially uniform, a development of striking epicardial dispersion of repolarization in AP can be seen [27].

Considering above reviewed researches, it is clear that, although they name dome or ND or SND in their models by some means and for different proposes, no one has analyzed or modeled dome, ND, or SND directly.

In this paper, we use a mathematical approach and present a new model for simulating biological phenomena, ND in ventricular AP. We consider agents involved in SND dynamics and propose simple yet sufficient principles and hypothesis theories for ND generation. As we will see, our mathematical idea will provide a new insight to the gating mechanism of ionic channels.

## 2. Method and Mathematical Approach

As we stated earlier, AP is the product of interactions among electrophysiological agents, most importantly and generally, activation and inactivation gating variables [1]. AP is a voltage that is generated across the cell membrane. AP is produced when interacting ionic currents flow through transmembrane ionic channels and charge membrane capacitance (see Figure 1(b)). Ionic channels can be opened and closed via gating process; gating process can be either activation (shown by *n*) or inactivation (shown by *h*). With the activation process, we mean a process that opens a channel more when membrane voltage increases. In inactivation process, a channel is closed more when membrane voltage increases.

A gating process can generate positive or negative feedback on membrane voltage. A positive feedback is created when activation variable, *n*, generates inward current or inactivation variable, *h*, generates outward current. In the same way, a negative feedback is created when activation variable, *n*, generates outward or inactivation variable, *h*, generates inward currents, respectively.

All of these mean that *n* and *h* can be generally considered two major interacting agents and mechanisms for AP generation. In this paper, we assume a general formalism for each ionic current as

(2) $$\begin{array}{c} I_{\text{ion}}=g_{\text{ion}}\cdot \left[\text{gating process}\right]\cdot \left(v-E_{\text{ion}}\right), \end{array}$$

where *g*_ion_ is the base conductance of ionic channel and [gating process] is a mathematical compound that is composed of gating processes; these processes may be voltage or time dependent or both.

During the plateau phase of AP (phase 2 in Figure 1(a)), we assume (or approximate) a general exponential decreasing (increasing) dynamics for *h* and an increasing (decreasing) one for *n* over considered time interval, as follows:

(3) $$\begin{array}{c} h\left(t\right)=e^{-t/\tau_{h}}, \end{array}$$

(4) $$\begin{array}{c} n\left(t\right)=1-e^{-t/\tau_{n}}. \end{array}$$

Now, if we assume a compound gating variable, *nh*, then the dynamics of *nh* can be written as:

(5) $$\begin{array}{c} nh\left(t\right)=e^{-t/\tau_{h}}\left(1-e^{-t/\tau_{n}}\right). \end{array}$$

And for the speed (time derivative) of *nh*, we have:

(6) $$\begin{array}{c} \dot{nh}=-\frac{1}{\tau_{h}}e^{-t/\tau_{h}}\left(1-e^{-t/\tau_{n}}\right)+\frac{1}{\tau_{n}}e^{-t/\tau_{h}}e^{-t/\tau_{n}}=-\frac{1}{\tau_{h}}e^{-t/\tau_{h}}+\left(\frac{1}{\tau_{h}}+\frac{1}{\tau_{n}}\right)e^{-t/\tau_{h}}e^{-t/\tau_{n}}. \end{array}$$

Taking *τ*_*h*_/*τ*_*n*_ = *α* or *τ*_*h*_ = *ατ*_*n*_, one has:

(7) $$\begin{array}{c} \dot{nh}=-\frac{1}{\tau_{h}}e^{-t/\tau_{h}}+\frac{1}{\tau_{h}}\left(1+\alpha \right)e^{-t/\tau_{h}}e^{-t/\tau_{n}}=\frac{1}{\tau_{h}}e^{-t/\tau_{h}}\left[\left(1+\alpha \right)e^{-t/\tau_{n}}-1\right]. \end{array}$$

Considering Equation (7) that is an amazing one in our approach, the sign of the speed of *nh* is determined by the expression in bracket. Note that the term *e*^−*t*/*τ*_*n*_^ in the bracket is ≤1; then, the sign of the speed of *nh* is influenced by *α*, the ratio of the speeds (time constants) of the gating processes *h* and *n*. If we name the time point at which the sign of the speed changes as *t*_*s*_, then we have:

(8) $$\begin{array}{c} \left(1+\alpha \right)e^{-t_{s}/\tau_{n}}=1\to e^{-t_{s}/\tau_{n}}=\frac{1}{1+\alpha }\to \frac{t_{s}}{\tau_{n}}=\ln\left(1+\alpha \right)\to t_{s}=\tau_{n}\ln\left(1+\alpha \right). \end{array}$$

Equation (8) that is a key equation in our research gives the time at which the sign of the speed of *nh* changes; i.e., an increasing (decreasing) dynamics evolves to a decreasing (increasing) one. It is clear from Equation (7) that, for *t* > *t*_*s*_, *nh* has an increasing dynamics, where for *t* < *t*_*s*_ has a decreasing one.

Now let us have a look at acceleration (second derivative) of *nh*. Using some differential mathematics manipulations, it can be shown that:

(9) $$\begin{array}{c} \dot{nh}=\dot{n}h+n\dot{h},\\ \ddot{nh}=\ddot{n}h+\dot{n}\dot{h}+\dot{n}\dot{h}+n\ddot{h} \end{array}$$

that leads to:

(10) $$\begin{array}{c} \ddot{nh}=-\frac{1}{{\tau_{h}}^{2}}e^{-t/\tau_{h}}\left[\left(1+\alpha \right)e^{-t/\tau_{n}}-1\right]-\frac{1}{\tau_{h}}e^{-t/\tau_{h}}\left(1+\alpha \right)\frac{1}{\tau_{n}}e^{-t/\tau_{n}}=-\frac{1}{\tau_{h}}e^{-t/\tau_{h}}\left[\frac{1}{\tau_{h}}\left(1+\alpha \right)e^{-t/\tau_{n}}-\frac{1}{\tau_{h}}+\frac{1}{\tau_{n}}\left(1+\alpha \right)e^{-t/\tau_{n}}\right]=-\frac{1}{{\tau_{h}}^{2}}e^{-t/\tau_{h}}\left[\left(1+\alpha \right)e^{-t/\tau_{n}}-1+\alpha \left(1+\alpha \right)e^{-t/\tau_{n}}\right]=-\frac{1}{{\tau_{h}}^{2}}e^{-t/\tau_{h}}\left[{\left(1+\alpha \right)}^{2}e^{-t/\tau_{n}}-1\right]. \end{array}$$

Considering Equation (10) that seems similar to Equation (7), it is interesting that the sign of the acceleration of *nh* is determined by the expression in the bracket. If we denote the time point at which the sign of acceleration changes, by *t*_in_, so we can write:

(11) $$\begin{array}{c} {\left(1+\alpha \right)}^{2}e^{-t_{\text{in}}/\tau_{n}}-1=0\to t_{\text{in}}=\tau_{n}\ln\left[{\left(1+\alpha \right)}^{2}\right]. \end{array}$$

In Equation (11), *t*_in_ is the time at which the acceleration of *nh* becomes zero; i.e., *t*_in_ is the inflection point of *nh*(*t*). In other words,*t*_in_ is a point at which the graph of *nh*(*t*) changes its curvature.

**Figure 2:**
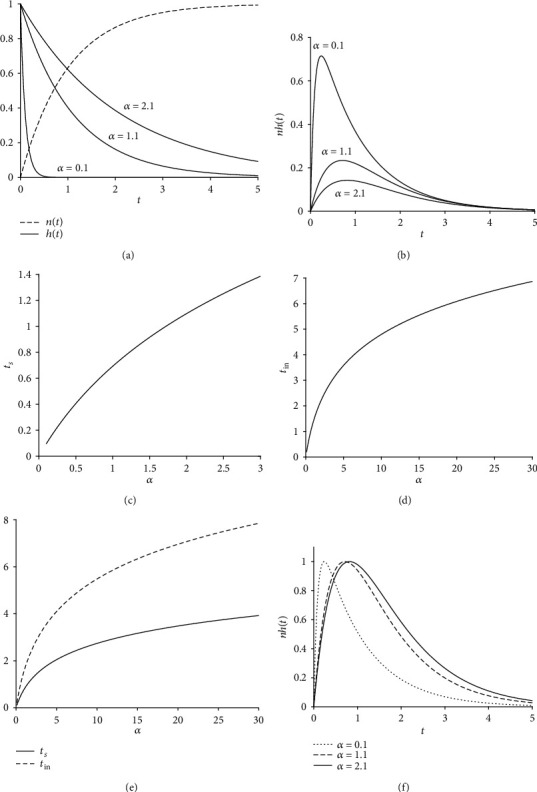
Graphical illustrations of (a) *n*(*t*) and *h*(*t*). (b) *nh*(*t*) for three different values of *α*. (c) *t*_*s*_ against *α*. (d) *t*_in_ against *α*. (e) *t*_*s*_ and *t*_in_ against *α*. (f) Scaling *nh*(*t*) so that it takes the maximum value of unit. In all panels, *τ*_*n*_ = 1 is fixed.

In Figure 2, we have illuminated the above mathematical discussions graphically.  Figure 2(a) illustrates sample graphs of *n*(*t*) and *h*(*t*), for three different values of *α*, where *τ*_*n*_ = 1 is fixed. It is clear that by increment of *α*, *h*(*t*) becomes slower in comparison with *n*(*t*). In Figure 2(b), we have sketched compound variable *nh*(*t*) against the time; it is seen that by increasing *α*, while the amplitude value of *nh*(*t*) decreases, its skewness (peak value) shifts to the right. Another interesting finding in Figure 2(b) is that for early times, the dynamics of *nh*(*t*) is like *n*(*t*), where for later times, i.e., after the peak of *nh*(*t*), the dynamics is similar to *h*(*t*). In Figure 2(c), the time point *t*_*s*_, i.e., the time at which the speed of *nh*(*t*) gets its maximum value, is plotted against *α*. In Figure 2(d), the inflection point of *nh*(*t*), *t*_in_, is plotted against *α*; it is clear that by increment of *α*, the position of *t*_*s*_ and *t*_in_ can be shifted to the right of the time axis; i.e., *t*_*s*_ and *t*_in_ happen at higher values of time.  Figure 2(e) depicts that *t*_in_ is always greater than *t*_*s*_; it happens after *t*_*s*_. Inspection of the slope of curves in Figures 2(c) and 2(d) makes it clear that *t*_in_ is more sensitive to the variations of *α* in comparison with *t*_*s*_. In fact, it will be so interesting if we use Equations (8) and (11) and get:

(12) $$\begin{array}{c} \frac{\partial t_{s}}{\partial \alpha }=\tau_{n}\frac{1}{1+\alpha }, \end{array}$$

(13) $$\begin{array}{c} \frac{\partial t_{\text{in}}}{\partial \alpha }=2\tau_{n}\frac{1}{1+\alpha }. \end{array}$$

Considering Equations (12) and (13), it is clear that *∂t*_in_/*∂α* = 2(*∂t*_*s*_/*∂α*) which means *t*_in_ is twice more sensitive to the variations of *α* than *t*_*s*_.

Substituting Equation (8) into Equation (5), it is easy to show that:

(14) $$\begin{array}{c} {nh}_{\max}=nh\left(t_{s}\right)=\alpha ∙{\left(1+\alpha \right)}^{-\left(\left(1+\alpha \right)/\alpha \right)}. \end{array}$$

So if we want to treat *nh* as an individual gating variable that can take a maximum value of unit, we can scale Equation (5) using Equation (14). In Figure 2(f), we have done so.

In Figure 2(e), we have sketched *t*_*s*_ and *t*_in_ on the same screen. It is seen that *t*_in_ is always larger than *t*_*s*_; i.e., the change in the curvature of *nh*(*t*) occurs after the maximum (minimum) speed. It is also evident that by increment of *α*, the distance between *t*_*s*_ and *t*_in_ in time domain increases.

Figure 2 clarifies how the value of *α* influences the dynamics and morphology of *nh*(*t*). As we will see later, the introduced novel parameter, *α*, plays a key role in our proposed formalism and model; i.e., one may use the above findings and facts for regulation of the AP morphology via the adjustment of the ratio of the speeds of activation and inactivation processes in a supposed system of ventricular cell.

**Figure 3:**
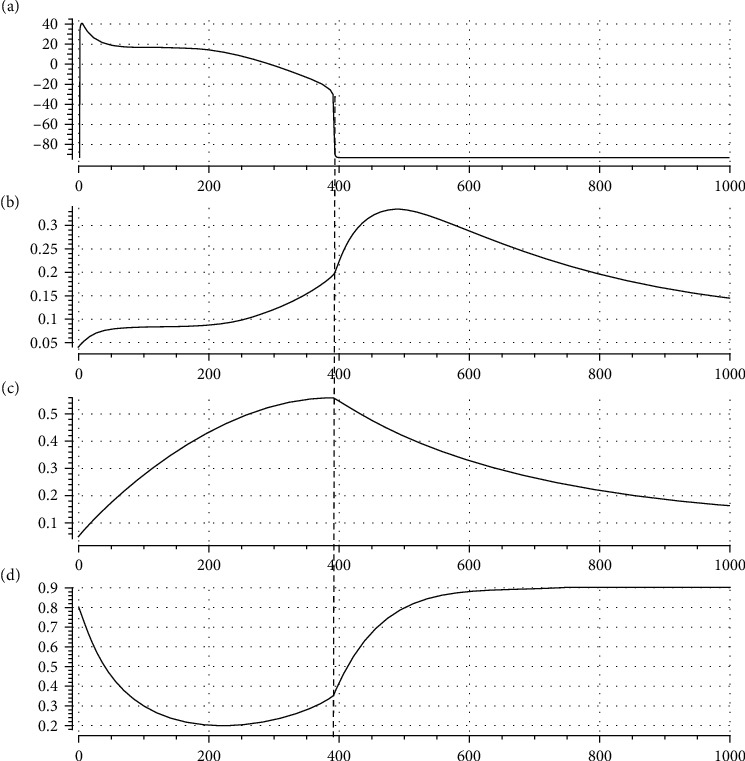
Typical realizations of (a) AP, (b) *nh*(*t*), (c) *n*(*t*), and (d) *h*(*t*), in a ventricular cell model. Here, *n*(*t*) and *h*(*t*) are the gating process. Compare these general morphologies with those in Figure 2.

Figure 3 depicts a typical simulation of AP, *nh*(*t*), *n*(*t*), and *h*(*t*) in a model of ventricular cell during the same time interval, where *α* is set to 0.65. You see how the evolutions of *nh*(*t*), *n*(*t*), and *h*(*t*) are similar to the general forms of Figure 2, particularly on the right side of the vertical dashed line where transmembrane potential, *v*, settles down toward rest potential; i.e., *v* has no variations. This figure will be more discussed in Results.

In this research, for explanation of our novel mathematical approach, i.e., illumination of time constant ratio hypothesis, we propose a mathematical model that is compatible with the biology of cardiac ventricular cell. Our model is composed of three currents: *I*_*L*_, a leakage current (linear component), *I*_*f*_, a dynamicless or fast current (nonlinear component) and *I*_*d*_, a nonlinear dynamical (slow) current. Following HH general formalism in Equation (1), the proposed model is defined in Equations (15), (16), (17) and (18) as follows:

(15) $$\begin{array}{c} \dot{v}=-\left(I_{L}\left(v\right)+I_{f}\left(v\right)+I_{d}\left(v,t\right)\right), \end{array}$$

where each current undergoes the general formalism of Equation (2) as:

(16) $$\begin{array}{c} I_{L}=g_{L}\cdot \left(v-E_{L}\right), \end{array}$$

(17) $$\begin{array}{c} I_{f}=g_{f}\cdot m\left(v\right)\cdot \left(v-E_{f}\right), \end{array}$$

(18) $$\begin{array}{c} I_{d}=g_{d}\cdot n\left(v,t\right)\cdot h\left(v,t\right)\cdot \left(v-E_{d}\right). \end{array}$$

We define the dynamics of activation, *n*, and inactivation, *h*, agents as follows:

(19) $$\begin{array}{c} \dot{n}=\frac{n\left(v,\infty \right)-n\left(v,t\right)}{\tau_{n}}, \end{array}$$

(20) $$\begin{array}{c} \dot{h}=\frac{h\left(v,\infty \right)-h\left(v,t\right)}{\tau_{h}}, \end{array}$$

(21) $$\begin{array}{c} n\left(v,\infty \right)=\frac{1}{1+\exp\left(\left(V_{12n}-v\right)/K_{n}\right)}, \end{array}$$

(22) $$\begin{array}{c} h\left(v,\infty \right)=\frac{1}{1+\exp\left(\left(V_{12n}-v\right)/K_{h}\right)}. \end{array}$$

The role of producing a regenerative process (positive feedback) for making an upstroke phase of AP has been assigned to *I*_*f*_ [43]. Note that we say nothing about the direction of *I*_*f*_; i.e., it may be treated as an inward or outward ionic current arbitrary. Here, we may assume a fast activation gating process, *m*(*v*), for *I*_*f*_ as follows:

(23) $$\begin{array}{c} m\left(v\right)=\frac{1}{1+\exp\left(\left(V_{12m}-v\right)/K_{m}\right)}. \end{array}$$

In the proposed model, *I*_*d*_ delegates all dynamical activation and inactivation agents; i.e., all voltage- and time-dependent positive and negative feedbacks on transmembrane voltage, *v*.

## 3. Results

**Figure 4:**
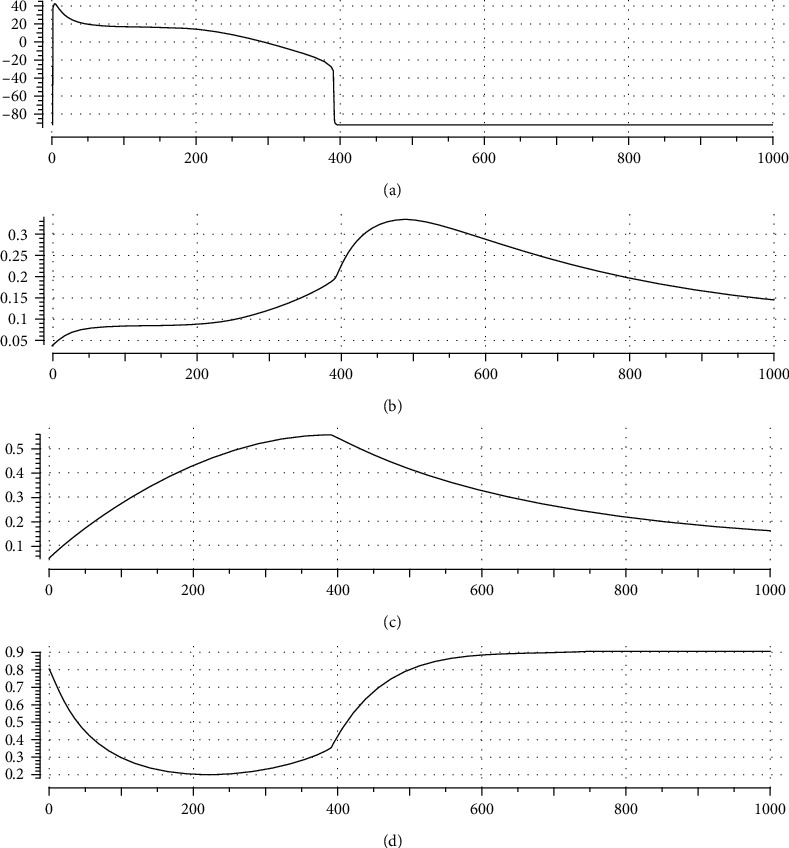
Evaluation of our proposed model for simulating ventricular cell AP: (a) AP morphology generated by the model; (b) time course of *nh*(*t*); (c) time course of *n*(*t*); (d) time course of *h*(*t*).

In this section, it is shown how AP morphology, particularly ND, that was obtained by complex electrophysiological models can be simulated by the presented model. Specially, it is shown how regulation of parameter *α*, i.e., adjusting the ratio of the activation speed to the inactivation speed, can regulate AP morphology. In the following investigations, we have used OpenCOR [44] and Matlab as simulation medium.  Figure 4 illustrates a typical simulation with the use of our model. It is seen that the proposed model can reflect the general morphology of ventricular AP satisfactorily.

The parameter values of the model for generating Figure 4 are listed in Table 1. In this table, we see that *E*_*f*_ > 0 means *I*_*f*_ acts as an instantaneous inward current and is responsible for generating the upstroke phase of AP (phase 0 in Figure 1(a)). We also have *E*_*d*_ < 0 that means *I*_*d*_ is an outward current and generally responsible for plateau phase shaping and AP repolarization. Here, because *I*_*d*_ is an outward current, activation and inactivation gating variables act as negative and positive feedbacks on membrane voltage, respectively.

**Figure 5:**
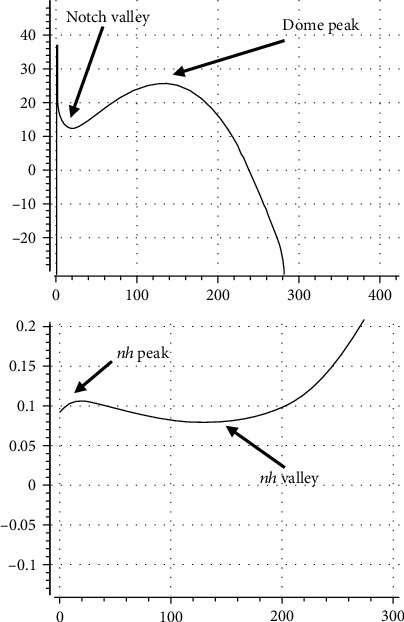
A zoomed-in view of SND of a simulated AP and its corresponding *nh*. This figure justifies our approach for regulating ND in a ventricular cell model.

For more clarification of our basic idea behind the proposed formalism for illustration of the role of *nh* as a compound gating variable, we consider Figure 5 that depicts a zoomed-in view of SND portion of a simulated AP and its corresponding *nh*. Because *I*_*d*_ is an outward current, its increment decreases membrane potential. It means that when *nh* gets its peak, we expect a minimum in membrane potential, i.e., a notch or valley. Similarly, when *nh* gets its minimum (valley), we expect a peak (dome) in AP. Comparison of time evolutions of AP and *nh* in Figure 5 justifies our approach for controlling notch and dome of an AP via regulating maximum and minimum of *nh*.

**Figure 6:**
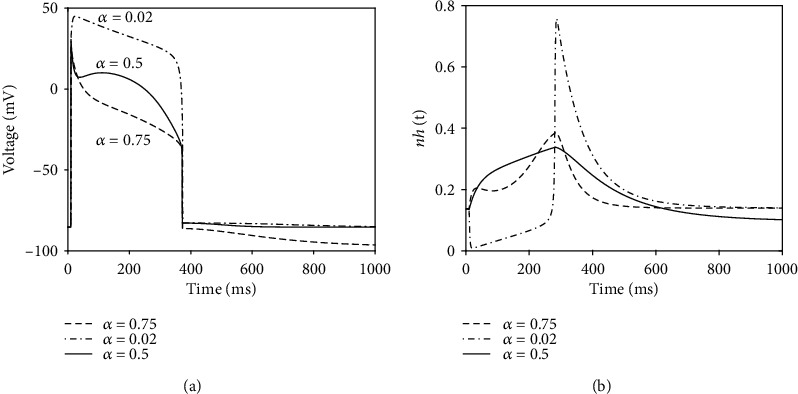
(a) Illustration of the effect of *α* on the general morphology of plateau phase of AP. We see how one can manage ND generation by regulating *α*. (b) Different morphologies of *nh*(*t*) generated by regulation of *α*. This figure validates graphically our hypothesis.

As we stated earlier, our new idea in this research is that the ratio of the speeds of activation and inactivation gating variables in a ventricular cell model can determine the morphology of AP; particularly, we may regulate ND in AP via regulation of *α*.  Figure 6 proves this idea by a graphical illustration. In Figure 6(a), we have sketched AP generated by our model for three different values of *α*. In Figure 6(b), the corresponding graphs of *nh*(*t*) are depicted. It is seen that the alteration of the ratio of activation and inactivation process speeds can affect AP morphology via alteration of *nh* dynamics.  Figure 6 also demonstrates how our approach is amenable to mathematical analysis and at the same time compatible with the electrophysiology of action potential; i.e., we can adjust AP morphology with the use of a biologically plausible parameter.

**Figure 7:**
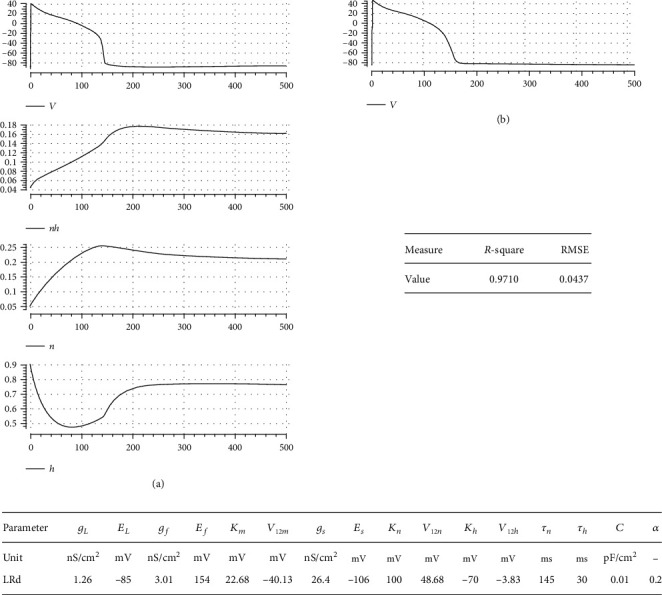
Fitting our model to the Luo-Rudy complex model: (a) simulation results: AP, *nh*, *n*, *h*; (b) AP of the Luo-Rudy complex model. Parameter value and goodness-of-fit measures are also reported in separate tables. The almost unit *R*-square shows how good our model can capture the morphology of AP.

**Figure 8:**
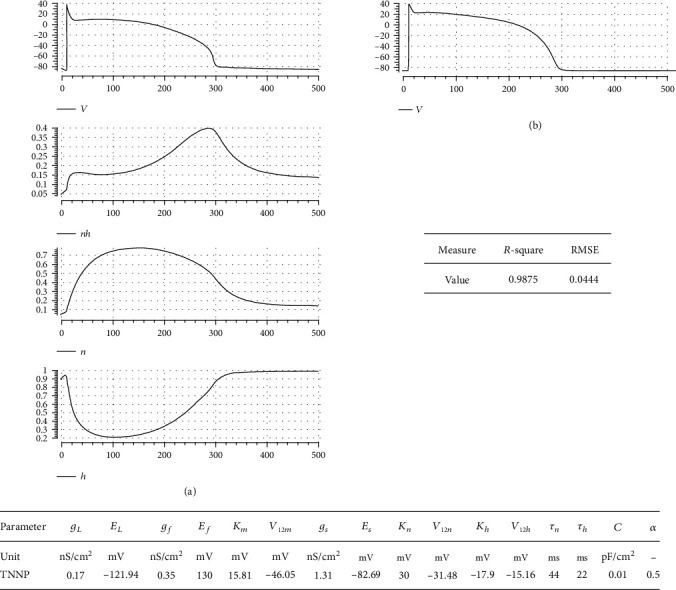
Fitting our model to Ten Tusscher et al. [2]: (a) simulation results: AP, *nh*, *n*, *h*; (b) AP of the Ten Tusscher et al. complex model. Parameter value and goodness-of-fit measures are also reported in separate tables. The almost unit *R*-square shows how good our model can capture the morphology of AP.

To show how our model and postulated activation to inactivation speed ratio approach can simulate well-known ventricular cell models, we fit it to two complex experimental data-based electrophysiological models: Luo and Rudy [9] and TNNP [2]. In Figures 7 and 8, simulation results are illustrated, respectively. In the left panel of each figure, simulated AP and gating variables, *nh*(*t*), *n*(*t*), and *h*(*t*), are sketched against time, where in the right one, the AP of the complex models is drawn. It is seen that our model can reflect general dynamics of these two models reasonably. The appended table in each figure lists the corresponding parameter value for the respective case. The goodness-of-fit measures are also reported in a separate table for each case. The nearly unit *R*-square shows how well our mathematical approach can simulate the dynamics of AP.

## 4. Discussion and Conclusion

In this research, we showed that our simple three-state variable model can simulate AP of ventricular cells. In addition, we revealed that our model can be fitted to well-known complex electrophysiological models. Our model was based on a novel idea that the ratio of the speeds of activation and inactivation gating variables in a ventricular cell model can govern the morphology of AP; particularly, we may regulate ND in AP via regulation of speed ratio. The results showed that our mathematical approach is useful to gain better biological understanding or explain simpler a biological phenomenon; AP.

The proposed speed ratio approach suggests a new way of looking at modeling and simulation of AP. The results demonstrated the effectiveness of our approach. We showed mathematically how the speed ratio coefficient, *α*, can influence the morphology of compound gating variable, *nh*, and how this gating variable which is used in an ionic current can affect membrane potential morphology by itself. In other words, speed ratio of activation and inactivation processes can be used as a tool for regulating AP morphology particularly ND shape. We see that *α* not only has an exact mathematical meaning but also has an exact and reasonable biological interpretation. From an electrophysiology point of view, while *α* actually delegates microscopic agents and players, it has great impacts on the macroscopic behavior, AP, of a cardiac cell.

As it was stated in Introduction, SND and particularly ND morphology have great impacts on cardiac arrhythmia and diseases. Therefore, we may treat *α* as a quantitative feature of the health of a ventricular cell. In this context, we may extend our idea to a broader area and hypothesize a novel impression about the quantification of the health of a neuron or cell in general. Following this idea, we may hypothesize that the health of a cell is influenced by the ratio of the speeds of positive and negative feedbacks on transmembrane voltage. We may also define a healthy range of *α* for any kind of cells, where with the healthy range, we mean an interval of *α* values on which the normal AP morphology can be created. This new idea should be more investigated in future researches.

Here, we should emphasize that we are biomedical engineers, so we look at biological systems from an engineering point of view. However, we have transactions with biologists and physicians in explaining our ideas and hypothesis. There are a huge number of examples that translating an idea from the engineering point of view to a biological one or vice versa has led to answer medical problems or to find therapy strategies. It means that sometimes, an engineering hypothesis which is mathematically satisfactory should be validated experimentally by a biologist. Similarly, an observation or finding by a biologist should be described mathematically by an engineer.

Experimental observations by physiologists have confirmed the following electrophysiological phenomena in the process of action potential generation [1]:

1. Inward ionic currents, depolarizing currents, for increasing membrane potential
2. Outward ionic currents, repolarizing currents, for decreasing membrane potential
3. Positive feedback on membrane potential for generating fast upstroke
4. Negative feedback on membrane potential for stability and rest potential generation
5. Activation and inactivation mechanisms of ionic channel gating process

Therefore, if the formalism of a mathematical model deals with the above biological phenomena, then it can be considered as a realistic biological model. In this circumstance, a finding or hypostasis that may be deduced from the model can be extended to the underlying biological system. However, this extension should be carried out by biologists and can be the subject of a separate biological research.

We have involved all above sensations in our model structure, so we can consider it an experimental-based model. Moreover, as we have shown in our paper, the proposed model can reproduce the AP morphology and dynamics of both Luo-Rudy and TNNP models, where Luo-Rudy and TNNP are based on experimental data [2, 9]. It means that our model which fits these models can be regarded as an experimental-based model as well. This look is also enhanced by the fact that our model has Hodgkin-Huxley- (HH-) based formalism.

Complex electrophysiological models of cardiac cell are sloppy [45]. With the sloppy, we mean models in which many parameters are loosely constrained and only depend on a few “stiff” parameter combinations. In a sloppy complex electrophysiological model, we expect that model behaviors can be controlled by a relatively small number of parameters and agent combinations. Among parameters and agent combinations, we should look for sets which deal with substantial concepts that influence complex system behaviors. In a ventricular cell, substantial agents are related to the abovementioned essential phenomena, i.e., ionic currents (inputs, outputs, and fast and slow with different underlying mechanisms) and gating process (activation and inactivation with different underlying mechanisms). These agents may induce positive or negative feedbacks on membrane voltage, where having at least one negative and one positive feedback on membrane voltage is necessary for AP generation [1]. We see that our model has representatives of all necessary electrophysiological agents in a ventricular cell: ionic currents, gating process, and positive and negative feedbacks.

Our presented and verified hypothesis states that complex morphology and dynamics of ND can be explained by a simple theory of the speed ratio (*α*) of activation and inactivation gating processes (*h* and *n*.). This statement suggests that the simple theories of macroscopic behaviors are hidden inside complicated microscopic processes which makes the complex world understandable [46].

Mathematical models of biological systems, which are mainly proposed by mathematicians or engineers, especially biomedical engineers, can play essential roles for clinical investigations, prediction of the disease behavior, and suggestion of suitable therapy protocols. In these circumstances, experiments and mathematical models are complementary. Biomedical engineers use experimental observations to propose appropriate framework for mathematical models, then benefit from the results of these models to improve future experiments. In this way and by using mathematical models, it will be possible for biologists to make predictions of situations that are difficult to implement in the lab. For example, electrophysiological mechanism such as gating speed cannot be easily measured experimentally. In this condition, one usually extrapolates experimental measurements. Considering today's experimental tools and methods, basic electrophysiological mechanisms cannot be easily measured by experiments. For example, patch clamp method which is one of the most common experiments usually suffers from interventions and effects of coupled cells. Also, no experimental techniques exist for measuring or controlling a single activation or inactivation gate speed. However, to show how our theory, the speed ratio (*α*) of positive and negative feedbacks through activation and inactivation effects, can describe the complex phenomenon, ND, we use widely used human ventricular cell by Priebe and Beuckelman [15] as a cell in laboratory and set up the following *in silico* experiment.

**Figure 9:**
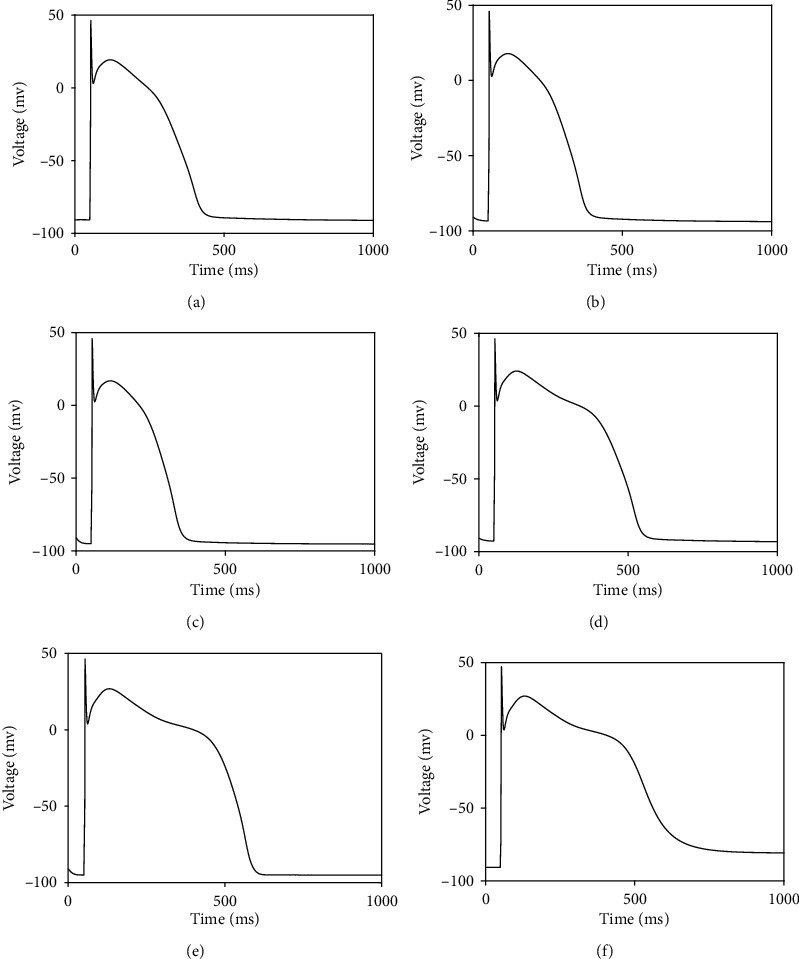
AP and ND morphology of PB cell where related ionic currents are present in a cell: (a) *I*_Na_ + *I*_Ca_ + *I*_to_ + *I*_Kr_ + *I*_Ks_ + *I*_K1_ + *I*_NaCa_ + *I*_NaK_ + *I*_bNa_ + *I*_bCa_ + *I*_stim_; (b) *I*_Na_ + *I*_Ca_ + *I*_to_ + *I*_Kr_ + *I*_Ks_ + *I*_K1_ + *I*_NaCa_ + *I*_NaK_ + *I*_bNa_ + *I*_stim_ (*I*_bCa_ is blocked); (c) *I*_Na_ + *I*_Ca_ + *I*_to_ + *I*_Kr_ + *I*_Ks_ + *I*_K1_ + *I*_NaCa_ + *I*_NaK_ + *I*_stim_ (*I*_bCa_ + *I*_bNa_ are blocked). (d) *I*_Na_ + *I*_Ca_ + *I*_to_ + *I*_Kr_ + *I*_Ks_ + *I*_K1_ + *I*_NaCa_ + *I*_stim_ (*I*_bCa_ + *I*_bNa_ + *I*_NaK_ are blocked); (e)  *I*_Na_ + *I*_Ca_ + *I*_to_ + *I*_Kr_ + *I*_Ks_ + *I*_K1_ + *I*_stim_ (*I*_bCa_ + *I*_bNa_ + *I*_NaK_ + *I*_NaCa_ are blocked); (f)  *I*_Na_ + *I*_Ca_ + *I*_to_ + *I*_Kr_ + *I*_Ks_ + *I*_stim_ (*I*_bCa_ + *I*_*bNa*_ + *I*_NaK_ + *I*_NaCa_ + *I*_K1_ are blocked).

Evoking the sloppiness of biological systems and in order to find the currents which are not explicitly involved in ND dynamics generation, we inhibit some currents in a cell. Results are summarized in Figures 9(a)–9(f). Each panel in Figure 9 depicts the general morphology of AP where the respective currents are inhibited.  Figure 9(f) illustrates the morphology of AP in the ventricular cell where a minimum number of currents are involved in its generation. In other words, these currents are necessary to reproduce AP and ND morphology qualitatively.

In Figure 9(f), we note that *I*_Ca_ is the only current that can create positive and negative feedbacks on membrane voltage simultaneously. This conception can be cleared mathematically if we have a look at *I*_Ca_ equation in PB:

(24) $$\begin{array}{c} I_{\text{Ca}}=g_{\text{Ca}}\cdot d\cdot f\cdot f_{\text{Ca}}\left(v-E_{\text{Ca}}\right), \end{array}$$

where *d* and *f* (and *f*_Ca_) are activation and inactivation gating variables, respectively.

**Figure 10:**
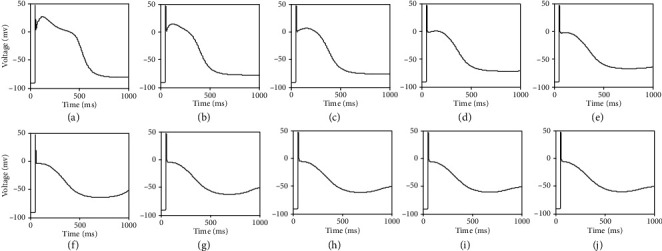
AP and ND morphology of PB cell where *α* is adjusted on a range of 1-10. Considering regulation of ND morphology with regulation of *α*, our hypothesis is validated experimentally.

In Figures 10(a)–10(j), we have validated our hypothesis by variation of *α* in a range of 1-10. You see how ND morphology can be biased by the speed ratio adjustment. The reader can also compare Figure 10 with Figure 6(a).

It is interesting that *I*_Ca_ is an inward current in PB cell. So its activation gate, *d*, creates a positive feedback and its inactivation gate, *f*, creates a negative feedback on membrane voltage. In a similar way, *I*_*d*_ is an outward current in our proposed model in which activation and inactivation gates generate negative and positive feedbacks, respectively. This fact shows a beautiful perspective of the generality of our hypothesis that speed ratio of positive and negative feedbacks can influence ND dynamics.

**Figure 11:**
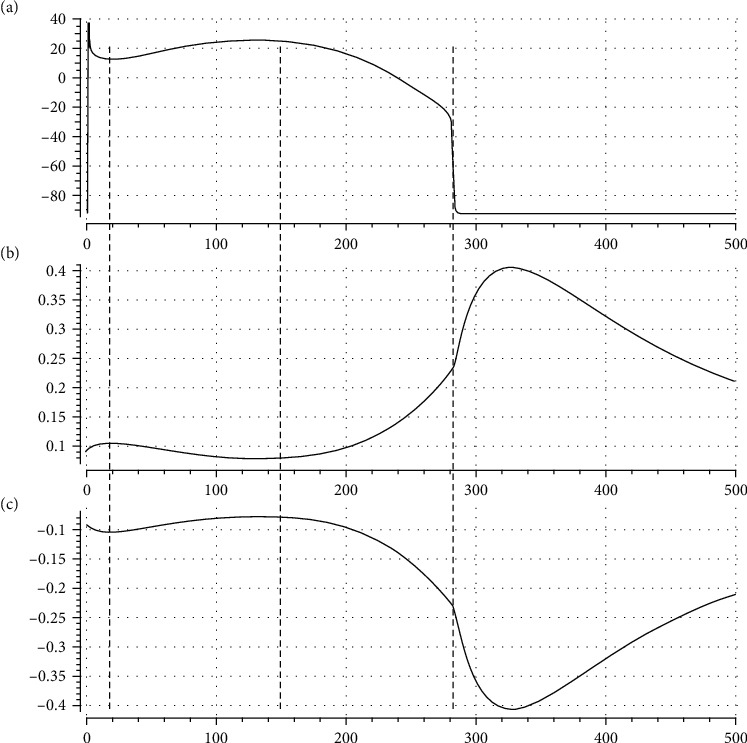
A typical simulation with the use of our model: (a) AP, (b) *nh*(*t*), and (c) reciprocal of *nh*(*t*) against time axis. We see how the dynamics of *nh*(*t*) reciprocal during the plateau phase is similar to AP.

Another amazing picture of our new idea is illustrated in Figure 11, where the two upper panels AP and *nh* are the same as in Figure 5 and the lower one is the flip of *nh* over the time axis; you see how the morphology of the plateau phase (the time evolution of AP after upstroke and before downstroke) is similar to the morphology of reciprocal of *nh*. The generalization of this interesting finding should be more investigated in future researches.

In the light of the speed ratio approach, some drug therapy strategies may be invented in the future, i.e., drug dosages that regulate the speed of activation and inactivation process of ionic channels.

The amenability of our approach to mathematical analysis makes it very suitable and flexible for applications in *in silico* drug therapy experiments and also introduction of a wide range of applications for it in cardiac arrhythmia simulations.

**Figure 12:**
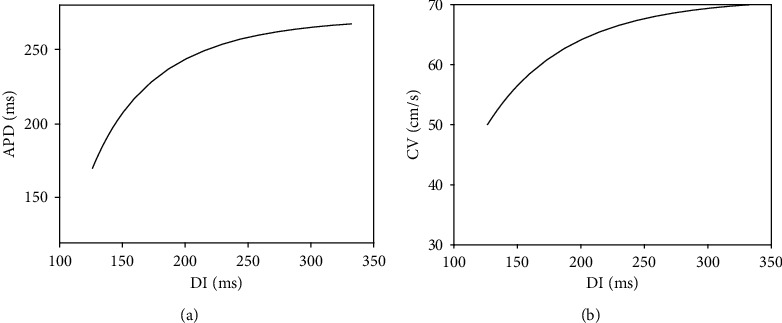
Sample restitution curves generated by our model: (a) APD restitution curve; (b) conduction velocity restitution curve. The proposed model and approach can simulate this important property of ventricular cells.

Although the impacts of speed regulation on AP morphology were investigated in this research, its impacts on other properties of AP dynamics, like AP propagation in tissue and restitution [47, 48], should be more investigated in future works. As a start point, we used our model with the parameter values in Table 1 where it generates an AP with ND. Then, we applied the dynamic restitution protocol [36] for driving action potential duration (APD) and conduction velocity (CV) restitution curves and the results are depicted in Figure 12. We see that our model can reflect generally the restitution property in ventricular cells [49].

## Figures and Tables

**Table 1:** Parameter value for generating AP in Figure 4.

Parameter	*g*_*L*_	*E*_*L*_	*g*_*f*_	*E*_*f*_	*K*_*m*_	*V*_12*m*_	*g*_*d*_		*E*_*d*_	*K*_*n*_	*V*_12*n*_	*K*_*h*_	*V*_12*h*_	*τ*_*n*_	*τ*_*h*_	*C*	*α*
Unit	nS/cm^2^	mV	nS/cm^2^	mV	mV	mV	nS/cm^2^		mV	mV	mV	mV	mV	ms	ms	pF/cm^2^	*—*
Value	0.01	-90	0.008	97.89	4.96	-38.1	0.072		-92.8	28.86	-29.4	-28.86	-29.4	300	65	0.01	0.22

## Data Availability

No data were used to support this study.
